# Optical Coherence Tomography Angiography in Branch Retinal Artery Occlusion

**DOI:** 10.4274/tjo.34270

**Published:** 2018-06-28

**Authors:** Tuna Çelik, Feyza Bilen, Fatime Nilüfer Yalçındağ, Huban Atilla

**Affiliations:** 1 Ankara University Faculty of Medicine, Department of Ophthalmology, Ankara, Turkey

**Keywords:** Acute vision loss, optical coherence tomography angiography, retinal artery occlusion, branch retinal artery occlusion

## Abstract

Optical coherence tomography angiography (OCTA) is a non-invasive alternative method used in the diagnosis and follow-up of acute branch retinal artery occlusion to show changes secondary to ischemia. We report a case with acute branch retinal artery occlusion. A 52-year-old man presented with a complaint of sudden-onset visual loss in the right lower quadrant of the left eye for the previous three days. Best-corrected visual acuity was 0.4 temporally. Inferonasal visual field deficit was detected with confrontation. Pupillary light reactions were normal in both eyes and there was no relative afferent pupillary defect. Dilated fundus examination revealed retinal lesion suggesting superior temporal branch retinal artery occlusion. He was treated with dextran 40 and pentoxifylline. Follow-up fundus fluorescein angiography could not performed because of chronic renal failure; OCTA demonstrated superficial and deep capillary non-perfusion areas and telangiectases in areas corresponding to the artery occlusion.

## Introduction

Acute retinal artery occlusion is an ocular emergency with painless, sudden-onset, unilateral loss of vision or visual field.^1^ Occlusion may occur at the level of the ophthalmic artery, the central retinal artery, a branch thereof, or the cilioretinal artery. It is more common in older men with cardiovascular disease.^2,3^ It is frequently associated with embolic or thrombotic diseases. Medical history and ophthalmologic examination are often sufficient for diagnosis but additional imaging methods may also be needed for diagnosis and follow-up. 

Fundus fluorescein angiography (FFA) is an invasive method requiring intravenous administration of dye that can cause side effects. In recent years, optic coherence tomography angiography (OCTA) has become widely available as an alternative to FFA in various ophthalmologic diseases. OCTA is a new non-invasive method for the detection and quantification of the retinal microcirculation without the use of dye but motion contrast. It senses erythrocyte movement in the vascular lumen by comparing the OCT signal amplitude between consecutive B-scans using the split-spectrum amplitude-decorrelation angiography algorithm, thereby providing high-quality vascular images with shorter acquisition times. Retinal tissue can be examined in layers via segmentation. Images from 3x3, 6x6 and 8x8 mm areas are used for the macula and 4.5x4.5 mm for the optic disc.^4,5^ It can quickly and non-invasively provide three-dimensional images of the retinal microvasculature.

## Case Report

**Figure 1:**
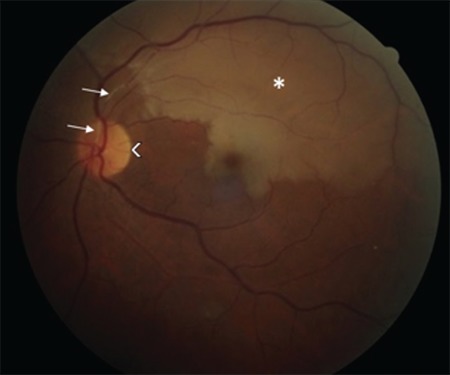
Color fundus photograph in the left eye shows sclerotic plaque in the proximal superotemporal artery and pallor of the superotemporal quadrant, macula and temporal optic disc

**Figure 2:**
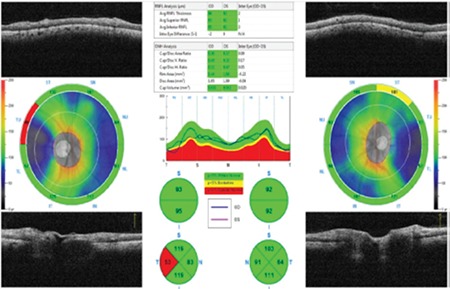
In optical coherence tomography images, the peripapillary retinal nerve fiber layer is within normal limits in both eyes OD: Right eye, OS: Left eye

**Figure 3:**
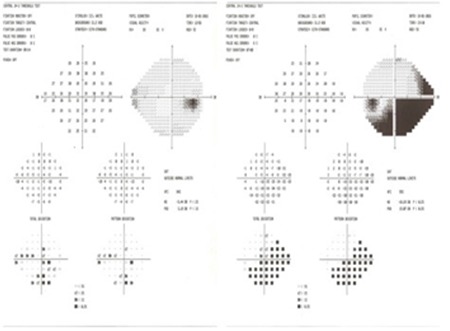
Threshold perimetry test shows loss of visual field in the inferior half of the left eye in accordance with superotemporal artery branch occlusion

**Figure 4:**
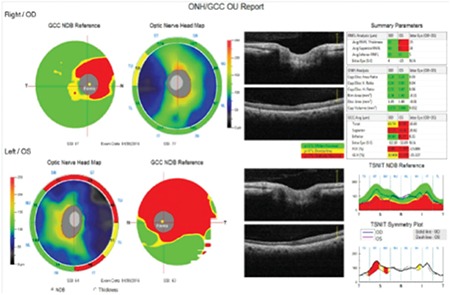
Peripapillary retinal nerve fiber layer thinning is apparent in the superior, inferior and temporal quadrants on optical coherence tomography OD: Right eye, OS: Left eye

**Figure 5:**
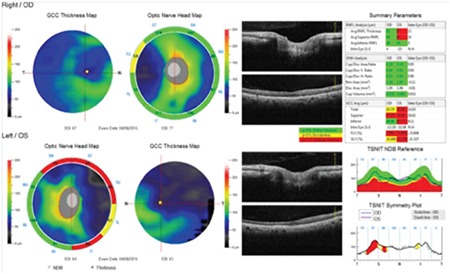
Optical coherence tomography thickness map indicated ganglion cell layer thinning in the superior and temporal quadrants OD: Right eye, OS: Left eye

**Figure 6:**
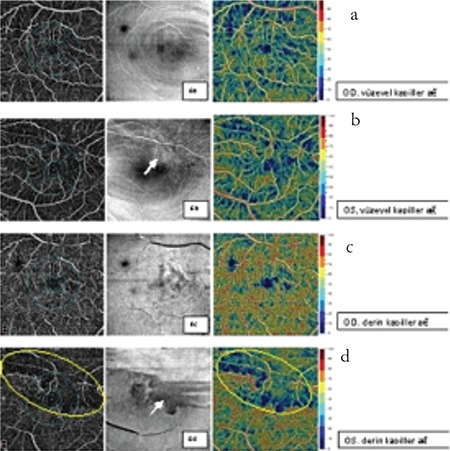
Macular optical coherence tomography angiography shows superficial capillary plexus loss and disruption of the deep capillary plexus in the left eye compared to the fellow eye. Ischemic areas are more clearly seen in en face images (b, d) OD: Right eye, OS: Left eye

**Figure 7:**
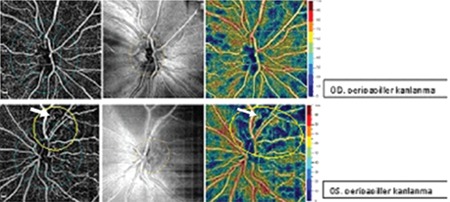
Optic disc optical coherence tomography angiography shows reduced papillary vascular density in the superotemporal optic disc and telangiectatic vessels (circles) in the left eye compared to the fellow eye OD: Right eye, OS: Left eye

**Figure 8:**
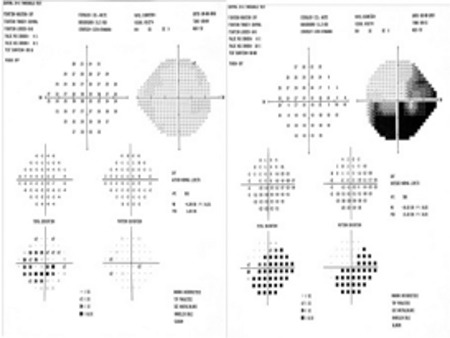
Visual field loss in the left inferior hemisphere persists in threshold perimetry after treatment

A 52-year-old male patient presented with the complaint of sudden vision loss in his left eye 3 days earlier. Past medical history was significant for chronic kidney disease, secondary hypertension, chronic hepatitis C virus infection and arrhythmia. Ophthalmologic examination revealed best corrected visual acuity of 10/10 in the right eye and 4/10 in the left eye from the temporal field. Confrontation test revealed inferonasal visual field loss in the left eye. Direct and indirect light reflexes were normal in both eyes and there was no relative afferent pupillary defect. Anterior segment examination was normal and intraocular pressure was 13 mmHg in both eyes. Dilated fundus exam demonstrated soft exudates consistent with hypertensive retinopathy in the right eye. Fundoscopy of the left eye revealed an area of pallor in the superotemporal quadrant and the macula with macular cherry red spot, which were consistent with occlusion of the superotemporal branch of the left retinal artery (Figure 1). On OCT, peripapillary retinal nerve fiber layer (RNFL) thickness was within normal limits (Figure 2). In the patient’s visual field, there was an inferonasal defect in the left eye corresponding to the occluded region (Figure 3). The patient was treated with a single dose of 500 cc intravenous dextran-40 and 200 mg intravenous pentoxifylline. In etiologic studies, Doppler ultrasonography revealed an atherosclerotic stenosis in the right and left main carotid arteries and a calcified plaque causing luminal narrowing in the left internal carotid artery. Transthoracic echocardiography revealed second- to third-degree aortic valve regurgitation and first-degree tricuspid valve regurgitation. There was no improvement in visual acuity or visual field despite treatment. At follow-up 7 months later, OCT showed thinning of the superior, inferior and temporal peripapillary RNFL (Figure 4). On the thickness map, ganglion cell layer was thinner in the superior and temporal areas (Figure 5). Decreased vascular density in the superficial and deep capillary plexus consistent with ischemia in the regions supplied by the superotemporal branch of the retinal artery was observed in a 6x6 mm macular field on OCTA (Figure 6). The borders of the ischemic area were more clearly seen in en face images (Figure 6b, d). In optic disc OCTA, capillary density was reduced in the superotemporal region and collateral vessels were present in the area (Figure 7). When compared to the fellow eye, there was a decrease in the macular deep and superficial capillary density in the superior and temporal quadrants (Table 1) and a decrease in peripapillary capillary density in the superior quadrant (Table 2). Visual field loss persisted in post-treatment threshold perimetry (Figure 8).

## Discussion

Acute branch retinal artery occlusion causes sudden, painless, unilateral, localized visual field loss in the retinal regions supplied by the affected artery.^6^ Although characteristic symptoms and fundus findings are sufficient for diagnosis of retinal artery occlusions, FFA can demonstrate lack of filling or slow filling of affected arteries along with completely normal choroidal perfusion. FFA has been utilized for approximately 50 years to visualize retinal vascular structures by injecting intravenous contrast agent. In the presented case, FFA could not be performed due to kidney disease. OCTA was recently introduced into clinical practice and is used in various retinal vascular diseases. An important advantage of OCTA is that it does not require the use of contrast agents. It can be safely used in patients with diseases limiting the use of contrast agents, such as kidney disease and those who require frequent follow-up. In retinal artery occlusion, edema resolves within weeks due to recanalization and reperfusion but vascular changes and atrophy of the inner retinal layers persist.^7^ Therefore, in cases where FFA is contraindicated, retinal morphology of the superficial and deep capillary plexus and the inner retinal layers can be visualized using OCTA with the help of its multilayer analysis. The ischemic area appears hyporeflective in en face OCTA imaging. We observed that in the superficial vascular plexus, not all collaterals were affected but some had disappeared, while there were areas of capillary drop-out and patchy areas of nonperfusion in the deep capillary plexus. In the literature it’s said that in branch retinal artery occlusion some capillaries may be dilated while others collapse.^8^ Our patient exhibited more pronounced ischemic areas and reduced capillary perfusion in the deep capillary plexus, consistent with the literature.^8,9^ The development of telangiectatic vessels was also observed in ischemic areas. Radial peripapillary capillaries were not detected in full-thickness analyses. OCTA images can be acquired in sizes of 3x3, 6x6, or 8x8 mm. Therefore, peripheral vascular lesions may not always be detectable in OCTA. When images are acquired in peripheral gaze position, the macula and optic disc are not included in the image area, thus limiting the use of the eye-tracking feature in the OCTA software and reducing image quality, thereby limiting vascular perfusion analyses. These limitations can be eliminated by using a montage technique or additional lenses for wide-angle imaging.^10,11^ There is no consensus regarding the timing and method of retinal artery treatment.^12,13,14^ Our patient presented 72 hours after the onset of symptoms and considering his initial visual acuity, we administered pentoxifylline and dextran therapy with the aim of increasing retinal tissue oxygenation via vasodilatation and hemodilution. Anticoagulant and antiaggregant drugs were used because of the patient’s comorbid conditions. Although clinical findings can be adequate for the diagnosis of branch retinal artery occlusion, imaging techniques such as FFA can be useful in differential diagnosis. In cases that have contraindication for FFA or other invasive techniques, new imaging modalities such as OCTA will be an effective and safe alternative in diagnosis and follow-up.

## Figures and Tables

**Table 1:**
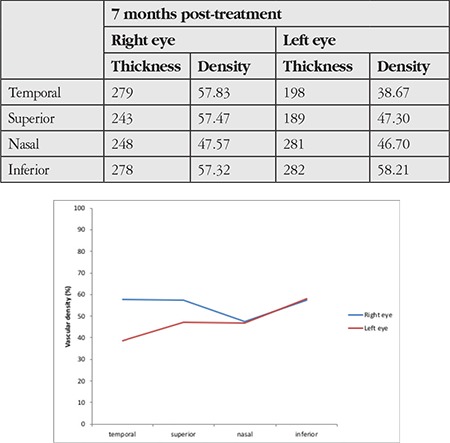
Comparison of superficial and deep vascular density in macular optical coherence tomography angiography images between the eyes showed a decrease in the superior and temporal quadrants of the left eye

**Table 2:**
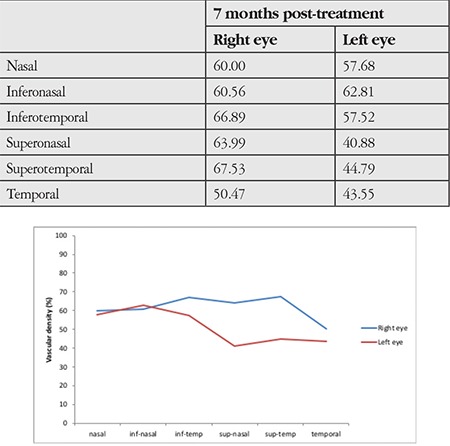
Comparison of peripapillary vascular density in optic disc OCTA images between the eyes showed a decrease in the superior quadrant of the left eye
